# Delineating the Role of Negative Verbal Thinking in Promoting Worry, Perceived Threat, and Anxiety

**DOI:** 10.1177/2167702615577349

**Published:** 2015-05-06

**Authors:** Colette R. Hirsch, Gemma Perman, Sarra Hayes, Claire Eagleson, Andrew Mathews

**Affiliations:** 1 Institute of Psychology, Psychiatry and Neuroscience, King’s College London; 2 Curtin University; 3 University of California, Davis

**Keywords:** anxiety, cognitive processes, threat

## Abstract

Worry is characterized by streams of verbal thoughts about potential negative outcomes. Individuals with high levels of worry (and particularly those with generalized anxiety disorder) find it very difficult to control worry once it has started. What is not clear is the extent to which verbal negative thinking style maintains worry. Our study aimed to disentangle the effects of verbal versus imagery based thinking, and negative versus positive worry-related content on subsequent negative intrusive thoughts. High worriers were trained to engage in imagery or verbal processing, focusing on either negative or positive outcomes of their current main worry. Both thinking style and valence of worry content influenced later negative intrusive thoughts that play a role in initiating worry episodes. In contrast, only valence influenced subjective ratings of worry outcomes (i.e., cost, concern, and ability to cope, although not probability), with positive valence leading to lower ratings, irrespective of thinking style.

We all worry from time to time, as if talking to ourselves about different ways in which things could go wrong, but are usually able to control worry if we need to focus on something else. In contrast, individuals with generalized anxiety disorder (GAD) report being unable to stop worrying, and data from experimental tasks confirms that they do indeed find it harder to control worry than matched high worriers without a diagnosis of GAD (Hirsch, Mathews, Lequertier, Perman, & Hayes, 2013). In contrast to other anxiety disorders (e.g., social anxiety disorder; posttraumatic stress disorder) that are characterized by aversive mental images, individuals with GAD experience imagery less frequently and those images that they do report are particularly brief (Hirsch, Hayes, Mathews, Perman, & Borkovec, 2012). Although this pattern is particularly marked in GAD, a similar predominance of quasi-verbal thought over imagery was observed when nonclinical participants are instructed to worry, in contrast to thinking about a positive future event. The reason for this domination of verbal thinking over imagery in worry (and particularly in GAD) is not entirely clear, although it has been suggested that it reflects attempts to resolve and avoid dangers, using typical (verbal) problem solving methods (Borkovec, Robinson, Pruzinsky, & DePree, 1983). However, many worries involve uncertain future threats that cannot be resolved in this way, and rather than being helpful, verbal encoding appears to lead to undesirable consequences contributing to difficulties in controlling worry (Hirsch & Mathews, 2012).

In previous experiments, when participants were instructed to worry in the usual manner (i.e., in verbal form) or to think about the same negative content in the form of mental images, the former instructions led to more frequent negative thought intrusions. For example, Butler, Wells, and Dewick (1995) asked nonanxious participants either to worry about a distressing film clip they had viewed, or to think about it in the form of mental images. Although the (verbal) worry group reported less anxiety at the time, they went on to have more negative thought intrusions about the film over the next few days. In more recent research with high worriers, instructions to worry about their main current worry topic in the usual verbal form increased subsequent negative thought intrusions compared with the baseline rate (preworry). In contrast, instructions to think about the same worry content in the form of images decreased later negative thought intrusions (Stokes & Hirsch, 2010). It is interesting that despite the intrusion differences, the high worry groups did not differ in terms of emotional state while thinking about their main worry topic in different ways. This is in keeping with other worry research that compares worry in imagery and verbal form (Leigh & Hirsch, 2011; Williams, Mathews, & Hirsch, 2014) but contrasts with the findings for nonanxious individuals who were told to think about an emotional film clip in different ways by Butler et al. (1995). Perhaps, because personal worry topics have by definition been thought about repeatedly on prior occasions, their emotional impact is difficult to modify. In contrast, the emotional impact of a novel film clip is likely to be more malleable, depending on how it is mentally represented. In any event, the dissociation between reported anxiety in chronic worriers and later intrusions suggests that other factors can be involved in maintaining intrusive thoughts.

There are a number of alternative explanations for the observed increase in later intrusive thoughts due to verbal processing in worry. As noted earlier, Borkovec and colleagues have proposed that verbal worry involves trying to resolve insoluble problems, resulting in the unresolved problems remaining more active in memory (cf. Zeigarnik, 1938). Similarly, Stöber (1998) suggested that—in contrast to the more specific concrete nature of images—worry content is typically more abstract and generalized. Thus chains of verbal thought about negative but vaguely specified events may prevent problems from being resolved and instead serve to maintain intrusions (see also Philippot, Baeyens, & Douilliez, 2006). In contrast, sustained imagery of worry content may enable habituation to specific imagined outcomes, or allow worriers to mentally “test out” the likelihood of concrete negative events, and draw on real-world experience that might challenge unrealistically negative outcomes. If so, then prolonged imagery could lead to the imagined situations being seen as less threatening or plausible, leading to fewer later intrusions with similar content.

An additional possibility is suggested by other comparisons of worry in verbal or imagined form, showing that verbal worry (but not imagery) interferes with simultaneous working memory tasks more than does thinking about positive topics, indicating that more attentional capacity is captured by verbal worry (Hayes, Hirsch, & Mathews, 2008; Leigh & Hirsch, 2011; Stefanopoulou, Hirsch, Hayes, Adlam, & Coker, 2014). Similarly, verbal worry leads to greater subsequent attentional bias to related threat cues than imagery (Williams et al., 2014). Both of these findings are consistent with the possibility that verbal worry is associated with greater attention to worry-related threat content, at least in high worriers, perhaps increasing the probability that subsequent negative intrusive thoughts will similarly capture attention and make it more difficult to ignore them.

If verbal processing per se does indeed promote intrusions and, by extension, the uncontrollability of worry, then interventions that encourage imagery-based processing of worry-related material should prove beneficial. As noted earlier, in one study, simply instructing worriers to think about the content of their worries in the form of mental images reduced later negative intrusions (Stokes & Hirsch, 2010). However, it is unclear whether verbal representation in itself is directly responsible for increasing later intrusive thoughts, or whether this effect depends at least partly on the effects of this type of processing on threat perception. If the general and abstract nature of verbal worry serves to enhance the overall perception of threat, this might explain why verbal worry takes up more limited attentional capacity (Leigh & Hirsch, 2011) and enhances subsequent attentional bias to threat (Williams et al., 2014). In contrast, because mental images tend to be more concrete and specific, they may be evaluated more realistically, allowing them to be perceived as less probable and perhaps also explaining the absence of subsequent attentional bias.

At first glance this might seem implausible, given the finding that representing novel events as mental images is typically reported to be more emotional than verbal descriptions (Holmes & Mathews, 2005; Mathews, Ridgeway, & Holmes, 2013). However, thinking about familiar personal worry-related outcomes using imagery in high worriers appears to be an exception to this rule (see Stokes & Hirsch, 2010; see also Leigh & Hirsch, 2011; Williams et al., 2014). It is not obvious why this should be so, but consistent with previous findings (e.g., Stöber, Tepperwien, & Staak, 2000), streams of verbally encoded thoughts about many possible negative outcomes may be semantically linked so as to promote a general sense of impending threat, in a way unlikely to occur when specific outcomes are represented as concrete images. If so, then substituting more positive content (whether in verbal or imaged form) for the typical stream of negative thoughts might reduce perceived threat and thereby decrease later negative intrusions. To investigate this issue we sought to contrast combinations of image or verbal-based thinking focused on either negative or positive worry-related content. In this way we aimed to test whether replacing typical verbal worry with either positive verbal content or with either negative or positive imagery would reduce later negative intrusions and perceived threat.

One possibility is that *any* imagery (regardless of its emotional content) will decrease intrusions relative to verbal worry, due to the relatively more concrete images blocking the habitual streams of negative verbal representations typical of worry. If so, then even negative imagery would be expected to reduce later intrusions in comparison with verbal worry (Hypothesis 1).

However, a second possibility is that imagery acts by reducing the *negativity* of worry content (for example, if the content of negative images is perceived as less plausible than similar verbal representations). If so, then replacing worry with unambiguously positive imagery may be more effective than negative imagery in reducing later intrusions, by introducing competing positive alternative outcomes (Hypothesis 2). Indirect support for this idea is provided by research into social anxiety disorder in which generating positive (rather than negative) self-images was found to reduce anxiety and improve social performance (Hirsch & Clark, 2007; Hirsch, Clark, Mathews, & Williams, 2003; Hirsch, Meynen, & Clark, 2004).

Although these two hypotheses can be seen as making competing predictions about whether modality of thought (imagery versus verbal) or emotional valence of image content (positive versus positive) should reduce later negative intrusions, they are not mutually exclusive and both may contribute to overall effects. For example, imagery of positive outcomes might be superior to images of negative content, because in addition to blocking abstract verbal worry, positive image content might simultaneously counter the negative meanings typically rehearsed in worry-related thought. If so, then negative imagery should reduce negative intrusions compared with (verbal) worry but be less effective in this respect than positive imagery (Hypothesis 3: both Hypothesis 1 and Hypothesis 2 are correct).

Alternatively, if we suppose that negative intrusions are caused mainly by involuntary recall of prior worry content, then they may be most effectively countered by practice in generating opposing positive meanings in the same (verbal) modality, because later intrusions in verbal form might be more likely to prime their competing (verbal) positive alternative. If so, then rehearsal of positive alternatives in verbal form should be more effective in reducing negative intrusions than imagery even if it is similarly positive in content (Hypothesis 4). Although these different considerations make it difficult to predict which precise combination will be most beneficial, we suppose that to the extent that imagery per se reduces intrusions, this should reflect a direct causal role for verbal processing, and correspondingly, the extent that substituting more positive meanings reduces intrusions should reflect the causal role of negative emotional content.

One possible mechanism that we have suggested may mediate the effect of worry on later intrusions is that verbal worry enhances perceived threat and makes it more likely that threat cues (or thoughts) will capture attention (Williams et al., 2014). To investigate this possibility, we assessed the perceived threat of worry content in several ways: via the subjective probability and cost of worry-related outcomes, degree of concern and perceived ability to cope with them, as well as current mood. We reasoned that, if replacing the usual form of worry with imagery or positive content decreases the ease to which negative outcomes come to mind (availability), this should be reflected in ratings of subjective probability, which are known to depend on use of the availability heuristic (MacLeod & Campbell, 1992). Similarly, if effects depend on the perceived severity of anticipated outcomes, this should be indicated by reductions in ratings of cost or ability to cope. As noted earlier, Stokes and Hirsch (2010) found no differences in mood over the worry period for their negative imagery and verbal conditions (although see Butler et al., 1995, for somewhat different findings). Because the evidence for mood effects due to worry in verbal or image form seems mixed, ratings were also obtained of current mood (anxiety, depression and happiness) to further explore this issue.

In summary, the current study was designed to disentangle the effects of thinking style (verbal vs. imagery) and emotional valence of content (negative vs. positive) on subsequent negative thought intrusions and perceived threat (encompassing subjective probability, cost, degree of concern and expectation of coping), together with exploratory ratings of mood. Participants were trained to engage in imagery or verbal processing, focusing on either negative or positive outcomes of their main worry topic. Effects of each condition were then assessed on negative thought intrusions, perceived threat, and mood during worry. It was predicted that engaging in negative verbal thinking would lead to more negative intrusions and higher ratings of threat than the alternative image-based or positive forms of thinking about worry topics.

## Method

### Design

High worriers were allocated to think about either negative or positive outcomes of a current worry in verbal or imagery form; thus there were four conditions: (a) negative verbal, (b) negative imagery, (c) positive verbal, and (d) positive imagery. Frequency of negative thought intrusions was assessed during an initial baseline and postworry phase, in both of which participants were instructed to focus on their breathing. Participants subsequently provided expanded descriptions of these thought intrusions to allow an assessor to categorize their valence. Ratings were obtained of levels of concern during the period of focusing on the current worry topic, and postworry topic phase ratings were made of perceived likelihood, cost and ability to cope with worry topic outcomes. Visual analog scales were administered at various points to assess mood.

### Participants

Participants were recruited from the local community via an advertisement placed in a local London newspaper and an online London networking website. Volunteers completed an initial screening Penn State Worry Questionnaire (PSWQ; Meyer, Miller, Metzger, & Borkovec, 1990), and those scoring 56 and above were invited to take part. Molina and Borkovec (1994) reported that a score of 56 fell one standard deviation below the mean for individuals diagnosed with GAD. Only those participants who still scored 56 or above on the day of testing were included in the analysis.

Participants were randomly allocated to one of four groups, subject to the constraint that the groups were matched on PSWQ, education, and gender. Four volunteers were excluded for scoring below 56 on the PSWQ, and 11 others were excluded after the experiment because they indicated on rating scales that they had engaged in the instructed thinking style (imagery or verbal) for less than 60% of the time, or they reported less than 60% of their thought content during the relevant phase was as specified (positive or negative). In all, 4 people were excluded from the positive imagery group, 5 from the negative imagery group, and 3 each from the positive and negative verbal groups. Chi-square analyses require more than 5 observations per cell, so separate chi-square tests were performed comparing the frequency of excluded participants in relation to valence or thinking style conditions. Comparison of excluded and included participants who engaged in positive versus negative mentation was not significant, χ^2^(1, *N* = 95) = 0.06, *ns*, φ = .02. There was also no difference between imagery and verbal groups, χ^2^(1, *N* = 95) = 0.51, *ns*, φ = .07. Excluded participants were replaced to ensure that the final sample consisted of 80 participants, with 20 assigned to each of the four groups.

There were 6 male and 14 female participants in each condition (except negative imagery, with 5 and 15, respectively), χ^2^(3, *N* = 80) = 0.18, *ns, V* = .05. Average age and years of education did not differ between groups, both *F*s < 1, η_*p*_^2^ < .01 see Table 1 for means and standard deviations. Similarly there were no differences on the PSWQ (*F* < 1, η_*p*_^2^ = .01), trait scores on the State-Trait Anxiety Inventory–Trait (STAI-T; Spielberger, Gorsuch, Lushene, Vagg, & Jacobs, 1983), *F*(1, 76) = 1.24, *ns*, η_*p*_^2^ = .05), or Spontaneous Use of Imagery Scale (SUIS; Reisberg, Pearson, & Kosslyn, 2003) scores, *F* < 1, η_*p*_^2^ = .02. In all, 15 participants met criteria for GAD on self-report (using the Generalized Anxiety Disorder Questionnaire; GAD-Q-IV; Newman et al., 2002) in the positive imagery group and 16 in the other three groups. Separate chi-square tests performed on frequency of GAD diagnoses comparing valence or mentation style (due to too few observations to compare four groups) indicated that positive and negative conditions did not differ, χ^2^(1, *N* = 80) = 0.07, *ns*, φ = .031, and similarly no difference was evident for mentation style conditions, χ^2^(1, *N* = 80) = 0.07, *ns*, φ = .031.

**Table 1.** Personal Characteristics for Each Group

Measure	Negative verbal	Negative imagery	Positive verbal	Positive imagery
Age	33.30 (9.76)	31.75 (8.85)	32.80 (12.61)	33.10 (9.85)
Education	15.10 (2.20)	15.10 (2.05)	15.00 (2.15)	14.55 (1.88)
PSWQ	68.70 (5.57)	70.45 (6.65)	69.33 (6.42)	69.61 (5.85)
STAI-T	58.21 (8.18)	60.99 (6.91)	56.97 (7.34)	60.65 (8.65)
SUIS total	41.60 (6.36)	41.60 (6.06)	39.60 (8.35)	39.55 (9.86)

Note: Values are means, with standard deviations in parentheses. PSWQ = Penn State Worry Questionnaire; STAI-T = State-Trait Anxiety Inventory–Trait; SUIS = Spontaneous Use of Imagery Scale.

### Materials

#### Emotional assessment instruments

##### GAD-Q-IV

The GAD-Q-IV (Newman et al., 2002) is a self-report diagnostic measure of GAD that has demonstrated good test–retest reliability (with high likelihood of remaining stable across time, χ^2^(1, *N* = 148) = 42.1, *p* < .001), convergent validity (*r* = .66 correlation with PSWQ) and discriminant validity (*r* = .34, for social anxiety as assessed by Social Interaction Anxiety Scale; Mattick & Clarke, 1998), and a high level of diagnostic agreement (*K* = .67) with a clinical assessor on the Anxiety Disorders Interview Schedule (Brown, DiNardo, & Barlow, 1994).

##### PSWQ

Trait worry level was measured using the PSWQ (Meyer et al., 1990), a 16-item measure consisting of statements about worry (e.g., “My worries overwhelm me”), each with a 5-point answer scale from 1 (*not at all typical of me*) to 5 (*very typical of me*), yielding a total score ranging from 16 to 80, with higher scores indicating greater worry levels. The PSWQ has good psychometric properties in student, community, and clinical samples, with studies reporting high internal consistency (.91), short-term retest reliability (.92), and convergent and criterion related validity (Brown, Antony, & Barlow, 1992; Davey, 1993; Meyer et al., 1990).

##### STAI-T

Trait anxiety was measured using the STAI-T (Spielberger et al., 1983), consisting of 20 anxiety symptoms participants rate for frequency of occurrence. Scores range between 20 and 80, with a higher score indicating greater anxiety. The STAI-T has good internal consistency (.89) and test–retest reliability (.88; Barnes, Harp, & Jung, 2002).

##### SUIS

Tendency to use imagery in everyday life was assessed using the SUIS (Reisberg et al., 2003), a 12-item measure comprising statements regarding use of imagery (e.g., “When I think about visiting a relative, I almost always have a clear mental picture of him or her.”). Participants rated each item on a scale ranging from 1 (*never appropriate*) to 5 (*always completely appropriate*). Reisberg et al. (2003) reported a mean score of 3.1 (range = 1.2–4.7) and internal consistency of *r* < .98. Test–retest reliability is not available for this measure.

##### Mood ratings

Three visual analog mood rating scales, each 10 cm in length, assessed current anxiety, depressed mood, and happiness with anchor points *not at all* at one end and *extremely* at the other. Participants placed a cross (x) on each scale, and scores were assigned by measuring its position, ranging from 0 (*not at all anxious/depressed/happy*) to 10 (*extremely anxious/depressed/happy*). These mood rating scales have been shown to have good construct validity, with anxious and depressed mood significantly increasing postworry as well as significantly decreasing from postworry to after the second breathing focus period, with significant changes in a converse direction on happiness ratings (Hirsch, Hayes, & Mathews, 2009). Good concurrent validity was also demonstrated in a sample of 20 community volunteers by significant positive correlations between the STAI-T (Spielberger et al., 1983) and anxiety (*r* = .66, *p* < .01), and depressed mood ratings (*r* = .45, *p* < .05), and negative correlations with happiness ratings (*r* = –.61, *p* < .01).

#### Worry topic task

This was adapted from the Worry Task (Hayes, Hirsch, & Mathews, 2010; Hirsch et al., 2013), used to assess thought intrusions following a period of instructed worry. The current version consisted of four phases: a 5-min baseline period in which participants were instructed to focus attention on their breathing, training in imagery or verbal thinking about positive or negative outcomes, a 6-min worry topic period of thinking in the designated manner, followed by a final 5-min breathing focus period.

During each breathing focus period, at 12 random intervals a tone indicated that participants should report if they were indeed focused on their breathing or having any other thoughts. If so, they categorized thoughts as positive, negative, or neutral and provided a brief label to describe its content. A Sony VAIO laptop computer running E-Prime software (Psychology Software Tools, Sharpsburg, PA) was used to present the tones during this task.

At the end of the breathing focus period participants were cued with each label in turn to provide a full description of what was going through their mind at the time. An Olympus WS-200S digital recorder was used to record participants’ expanded descriptions for later rating by independent assessors, who had no knowledge of the participants’ assigned condition or time of breathing focus (i.e., baseline or postworry topic). One assessor rated the valence of all thought intrusions as positive, neutral, or negative, and a second categorized a third of the sample to allow a reliability check. Interrater reliability (Cohen’s κ) for valence ratings was .84.

### Procedure

Participants first completed informed consent and the emotional assessment questionnaires, and carried out the baseline breathing focus task, before being assigned to their designated form of training.

Participants in the imagery groups were first asked to think about “friendship” in mental images, whereas the verbal groups thought about it in words and sentences. They then completed separate ratings of how much of their thinking was either in imagery or verbal form (0%/not at all, to 100% all the time). Participants were then presented with six potentially worrying scenarios that covered a range of domains. The negative groups (imagery or verbal) practiced thinking about negative outcomes of each scenario in their designated style, whereas the positive groups thought about positive outcomes. The amount of time they thought about each scenario was progressively increased (1 min for Scenario 1, 1.5 min for Scenarios 2 and 3, and 2 min for the final three scenarios). Participants rated their thinking style after each scenario, and the percentage of their thoughts that were positive, negative, or neutral.

After the designated training, all participants identified their main current worry topic, and the experimenter prompted them to identify negative outcomes of the worry (“What are some of the bad things about it?” “What would be the worst possible outcome?” “What would be bad about that?”). The experimenter noted down the participant’s main negative outcome.

After identifying negative outcomes, the positive groups only were required to identify alternative positive outcomes of the worry topic, prompted by questions such as “What are some of the positive outcomes of this?” “How might it turn out OK?” and “What would be positive about that?” All participants were then asked to focus on their positive or negative outcomes (depending on group allocation) in their designated style (imagery or verbal) for 2 min. They then rated how concerned they were about their worry topic on a scale from 0% (*not at all*) to 100% (*extremely*). Participants then continued thinking about their identified topic in the designated manner, followed by ratings as before, for two more 2-min periods.

Participants were then asked to engage in a second breathing focus period for 5 min, as described earlier. When this was complete, they were reminded of the previously reported negative outcome of their worry topic and rated the likelihood of this happening to them (i.e., probability) and how bad it would be if it did happen (i.e., cost) using scales from 0 (*not at all*) to 100 (*extremely*). They also rated how well they would cope with the negative outcome if it happened on a 0 (*extremely well*) to 100 (*not at all well*) scale. Participants were then asked to cast their mind back to the prior breathing focus periods and prompted to recall in more detail what had been going through their minds for each reported intrusion. They were then asked to make retrospective ratings of thinking and emotional valence for each of the three periods in which they had been instructed to maintain their designated style. Finally, participants were asked what they thought the study had been about, and after their ideas had been recorded, were informed of the main hypotheses and asked if any thoughts along those lines had occurred to them. Participants were paid £15 ($24).

To ensure that the groups did not differ in terms of how negative their worry topic was, an assessor rated it as low, moderate, or highly negative, and a third of the topics were also rated by another assessor to check for reliability. Cohen’s kappa for interrater reliability was .92.

## Results

### Negative intrusions

A mixed-model ANOVA was conducted on the number of negative thought intrusions during the breathing focus periods, with within-subjects factors of time (baseline vs. postworry topic) and rater (self vs. assessor) and between-subjects factors of valence (positive vs. negative) and thinking style (imagery vs. verbal).

This revealed a significant main effect of rater, *F*(1, 76) = 5.44, *p* < .05, η_*p*_^2^ *=* .07, with participants reporting more negative intrusions than the assessor (see Table 2 for means and standard deviations.). However, rater did not significantly interact with any other factor (all *p >* .19, η_*p*_^2^ < .02), so this difference is unlikely to influence other effects. All subsequent analyses were performed on the average number of negative intrusions for self and assessor ratings. There was a significant main effect of time, *F*(1, 76) = 16.38, *p* < .001, η_*p*_^2^ = .18, reflecting the fact that negative intrusions reduced overall from baseline to postworry topic phase. There was also a nonsignificant trend toward a main effect of valence, *F*(1, 76) = 3.86, *p* = .053, η_*p*_^2^ = .05, but no main effect due to thinking style, *F*(1, 76) = 0.00, *p* = .99, η_*p*_^2^ = .00, nor any interaction of valence by thinking style, *F*(1, 76) = 0.00, *p = .99*, η_*p*_^2^ = .00, or thinking style by time, *F*(1, 76) = 0.95, *p* = .33, η_*p*_^2^ = .01. More important, two interactions were significant: a 2-way interaction between valence and time, *F*(1, 76) = 12.97, *p* < .001, η_*p*_^2^ = .15, that was qualified by a 3-way interaction between valence, thinking style, and time, *F*(1, 76) = 7.33, *p* < .05, η_*p*_^2^ = .09.

**Table 2.** Mean Number of Negative Thought Intrusions for Groups at Baseline and Postworry Topic Phase, as Rated by Self and Assessor

	Baseline		Postworry	
Group	Self	Assessor	Self	Assessor
Negative verbal	2.20 (1.77)	1.75 (1.55)	2.85 (2.52)	2.55 (2.04)
Negative imagery	2.85 (2.50)	2.75 (2.27)	1.95 (2.11)	1.80 (1.67)
Positive verbal	2.80 (2.44)	2.65 (2.54)	0.75 (0.85)	0.50 (0.89)
Positive imagery	2.55 (1.93)	2.10 (1.86)	1.00 (1.03)	1.00 (1.03)

Note: Values are means, with standard deviations in parentheses.

To better interpret these complex interactions we computed indices of change in negative intrusions over time (baseline—postworry), such that greater reductions would result in higher positive scores. These indices were then used to test each of the hypotheses discussed in the introduction.

*Hypothesis 1:* Did negative imagery reduce intrusions compared with negative verbal worry?

An independent sample planned comparison *t* test showed that negative imagery significantly reduced intrusions in comparison to negative verbal worry, *t*(38) = 2.65, *p* = .012, = 0.86 (0.93 vs. –0.73), thus supporting the first hypothesis and confirming the findings of Stokes and Hirsch (2010).

*Hypothesis 2:* Was positive imagery more effective in reducing intrusions than negative imagery?

Although positive imagery significantly reduced negative intrusions compared with verbal worry, a specific test of Hypothesis 2 failed to reveal a significant further reduction due to positive imagery, *t*(38) = 0.68, *p* = .50, *d* = 0.22 (1.33 vs. 0.93). The second hypothesis, that positive valence might further enhance the effects of imagery, was thus not supported.

*Hypothesis 3:* Was there evidence for additive effects of imagery and positive content?

As noted earlier, despite the finding that imagery (negative or positive) reduced negative intrusions relative to verbal worry, the difference between positive and negative imagery failed to reach significance. Hypothesis 3, that positive valence would add to effects of imagery per se, was thus similarly not supported.

*Hypothesis 4:* Did rehearsal of positive alternatives in verbal rather than imagery form further reduce negative intrusions?

Although the mean reduction of negative intrusions following positive verbal alternatives was numerically the largest (and significantly greater than after negative imagery), in the planned test of Hypothesis 4, there was no significant difference between verbal processing and imagery when both were positive, *t*(38) = 1.20, *p* = .24, *d* = 0.39 (2.10 vs. 1.33). The hypothesis that positive verbal processing might be particularly effective (and more so than positive imagery) in countering intrusions following verbal worry was thus not supported.

In summary, all alternative conditions were superior to the negative verbal style that is typical of worry in reducing negative intrusions, indicating that all of the alternative styles investigated here were helpful in reducing negative intrusions, without establishing clear evidence of differences among these alternatives.

### Subjective probability, cost, and ability to cope with potential negative outcome of the worry

Two-way ANOVAs with between-subjects factors of valence (positive vs. negative) and thinking style (imagery vs. verbal) were conducted separately on rated subjective probability (i.e., likelihood), cost (i.e., how bad it would be) and ability to cope with the negative outcome of the worry (see Table 3 for means and standard deviations). For subjective probability ratings, there was no main effect of valence, *F*(1, 76) = 0.34, *p* = .56, η_*p*_^2^ *=* < .01, thinking style, *F*(1, 76) = 0.00, *p* = 1.00, η_*p*_^2^ < .01, or a significant interaction between them, *F*(1, 76) = 0.00, *p* = .97, η_*p*_^2^ < .01.

**Table 3.** Mean Probability, Cost, and Coping Ratings for Worry Topic for Positive and Negative Conditions

Measure	Positive groups	Negative groups
Probability	6.05 (2.56)	6.35 (1.91)
Cost	7.00 (2.71)	8.26 (1.72)
Coping	5.74 (2.51)	7.16 (2.19)

Note: Values are means, with standard deviations in parentheses. High scores indicate greater probability and cost, and poorer coping.

In contrast, a significant main effect of valence emerged in the analysis of perceived cost, with positive conditions resulting in lower cost ratings than negative, *F*(1, 76) = 6.01, *p* < .05, η_*p*_^2^ = .07 (7.00 vs. 8.26). However, neither the main effect of thinking style, *F*(1, 76) = 0.26, *p* = .61, η_*p*_^2^ < .01, nor the interaction with valence was significant, *F*(1, 76) = 0.001, *p* = .98, η_*p*_^2^ = .00. Similarly, in the analysis of perceived ability to cope, the main effect of valence was significant, *F*(1, 76) = 7.08, *p* < .01, η_*p*_^2^ = .09 (positive *M* = 5.75, *SD* = 2.51 vs. negative *M* = 7.16, *SD* = 2.19), but neither the main effect of thinking, *F*(1, 76) < 0.01, *p* = .94, η_*p*_^2^ < .01, nor the interaction between them was significant, *F*(1, 76) = 0.95, *p* = .33, η_*p*_^2^ = .01.

In sum, these findings show that after thinking about positive outcomes of their worries participants anticipated that negative outcomes will be less costly (although no less likely), and that they would be better able to cope with them, compared with thinking about negative outcomes, whether in verbal or image form.

### Concern and mood ratings

The extent to which participants said that they felt concerned about their worry following each 2-min worry topic phase was examined in a mixed-model ANOVA with two between-groups factors, valence (positive vs. negative) and thinking style (imagery vs. verbal), and one within-subject factor of time (Periods 1–3). There was a main effect of thinking style, *F*(1, 76) = 7.53, *p* < .01, η_*p*_^2^ = .09, with imagery leading to lower concern ratings than verbal processing (68.60, vs. 80.35). There was also a main effect of time, *F*(1, 76) = 54.02, *p* < .001, η_*p*_^2^ = .415, and valence, *F*(1, 76) = 40.78, *p* < .001, η_*p*_^2^ = .35, which was qualified by a significant interaction between valence and time, *F*(1, 76) = 53.27, *p* < .001, η_*p*_^2^ = .41. As can be seen from Table 4, this is due to a reduction in concern over time for the positive conditions, *F*(1, 39) = 66.78, *p* < .001, η_*p*_^2^ < .63, but no such reduction in the negative conditions, *F*(1, 39) < 0.01, *p* = .95, η_*p*_^2^ < .01.

**Table 4.** Mean Worry Concern Ratings Over Time for Positive and Negative Conditions

Measure	Positive conditions	Negative conditions
Worry topic Phase 1	71.68 (25.14)	88.50 (13.72)
Worry topic Phase 2	60.35 (27.27)	87.53 (14.02)
Worry topic Phase 3	50.38 (26.38)	88.43 (14.50)

Note: Values are means, with standard deviations in parentheses.

Similar ANOVAs were conducted on anxiety, depression, and happiness ratings obtained after each of the three worry topic periods. For anxiety ratings, there were no significant effects involving thinking style, but main effects of time, *F*(1, 76) = 5.22, *p* < .01, η_*p*_^2^ = .06, and valence, *F*(1, 76) = 48.59, *p* < .001, η_*p*_^2^ = .39, qualified by an interaction between them, *F*(1, 76) = 9.16, *p* < .001, η_*p*_^2^ = .11. Simple planned follow-up *t* tests indicated that anxiety progressively decreased in the positive conditions from the first to the second, *t*(39) = 3.95, *p* < .001, *d* = 0.90 (3.97, vs. 3.29), and from the second to the final period, *t*(39) = 2.63, *p* < .05, *d* = 0.59 (3.29 vs. 2.73). There were no such effects in the negative groups.

Depressed mood ratings showed the same pattern, the most relevant being a significant interaction of valence and time, *F*(1, 76) = 6.82, *p* < .001, η_*p*_^2^ = .08. Simple planned follow-up *t* tests indicated a decrease from the first to second worry period in the positive conditions, *t*(39) = 2.11, *p* < .05, *d* = 0.47 (2.99 vs. 2.52), but no change from the second to final period, *t*(39) = 0.42, *p* = .67, *d* = 0.10, nor for any of the negative conditions.

Furthermore, happiness ratings during the worry topic phases produced a significant interaction between valence and time, *F*(1, 76) = 12.28, *p* < .01, η_*p*_^2^ = .14, with simple planned comparisons showing happiness increased in the positive conditions from the first to the second worry period, *t*(39) = 3.45, *p <* .01, *d* = 1.12, but not significantly between second and final periods, *t*(39) = 1.36, *p* = .18, *d* = 0.44. In relation to the negative condition, there was a nonsignificant trend for a reduction in happiness from time first to second worry periods, *t*(39) = 1.82, *p* = .07, *d* = 0.58, and no change between the second and final periods, *t*(39) = 0.07, *p* = .95, *d* = 0.02.

In summary, only the positive conditions resulted in mood changes during the worry topic phase, specifically a decrease in both anxiety and depression and an increase in happiness. These changes in mood were independent of thinking style because no main effects or interactions with modality were found.

To check whether these results could be attributed to differences in how negative the chosen personal worry topics were, assessors’ ratings of negativity for these topics were analyzed, but no significant group difference was found (χ^2^ = 2.96, *df* = 3, *p* = .43, *V* = .19).

## Discussion

The main findings of the present study can be summarized as showing, first, that intrusive thought frequency was influenced both by thinking style (imagery rather than verbal) and valence of content (positive rather than negative). Use of imagery, even when combined with negative content, reduced later intrusions relative to verbal worry, replicating Stokes and Hirsch (2010), and providing support for the hypothesis that verbal representation in worry has a role in maintaining negative intrusions.

Despite the apparent further reduction due to combining imagery with positive content, the difference from negative imagery failed to reach significance, so the hypothesis that the two effects would be additive was not supported. However, imagery per se does not seem to be essential for reducing intrusions, because thinking in *verbal* form about positive worry-related outcomes also significantly reduced mean intrusion frequency well below the level following verbal worry. Despite this finding, the difference between intrusions following image- or verbal-based positive conditions was not significant, so the hypothesis that thinking about positive outcomes in verbal rather than in imagery form might be especially effective in countering worry was not supported. Rather, the only firm conclusion possible is that all of the alternatives examined reduced intrusion frequency below that seen following worry as usual.

The second main finding was that subjective ratings of worry outcomes (e.g., of cost, concern, and ability to cope, although not probability) were also significantly reduced, but only when worry negativity was directly modified by substituting more positive content, with no significant differences according to whether positive thinking was in verbal or image form. The hypothesis that the beneficial effects of (negative) imagery on intrusions might depend on reducing the subjective threat value of worry content was thus not supported by ratings of worry content. Indeed, thinking style (verbal vs. imagery) did not significantly influence any of the indices of threat perception, which were lower only in groups required to generate more positive outcomes for worry-related concerns.

Assessors’ ratings of the negativity of the selected worry topics did not differ across groups, so that these findings cannot be easily attributed to preexisting differences in the worry topic itself. Unexpectedly, the subjective probability of negative outcomes did *not* differ significantly according to valence of content, arguing against the hypothesis that effects on rated threat value or intrusions were mediated by availability. Rather, it seems likely that the generation of positive outcomes decreased the perceived negativity of the expected outcomes themselves, as reflected in ratings of cost, concern, and coping, and this is one process whereby the frequency of intrusions perceived as negative can be reduced.

Although reduced negativity might help to account for a reduction in number of intrusions judged to be negative in the two positive groups, the question remains of why imagery per se (even when negative) also reduced negative intrusions because imagery did not significantly decrease any of the indices of threat perception. Anxious and depressed mood was also reduced only in groups rehearsing positive content, with no significant differences between imagery and verbal thought in this respect (replicating Leigh & Hirsch, 2011; Stokes & Hirsch, 2010; Williams et al., 2014). If there were no differences in mood or threat perception due to imagery per se, it seems that the capacity of imagery to reduce intrusions must depend on a different process.

One candidate explanation is that verbal processing, unlike imagery, simultaneously primes a wide range of semantically related potential threats, and so increases the range of possible negative thoughts that can intrude later (for a discussion of this possibility, see Hirsch & Mathews, 2012). As noted earlier, in high worriers (and particularly in GAD), habitual worries about many different potential negative outcomes could became semantically linked in memory, so that one worry primes another, and so on. This interpretation is consistent with the observation that thinking during worry seems relatively general or abstract, and could also explain why (verbal) worry leads to attention being captured by a wide variety of threat cues (Williams et al., 2014). By this explanation, imagery is important mainly by blocking the adverse consequences of verbal processing, especially those due to priming many different potential threat meanings. Such a possibility must remain speculative, but can readily be tested in future by assessing the capacity of verbal worry to prime a wider range of threat meanings than imagery.

At first glance, the absence of group differences in probability estimates seems inconsistent with the other differences found for threat perception. However, the worry topic was selected to be the person’s main current worry, so that by definition participants would have worried about it repeatedly. Consequently, the topic may remain highly available in memory and probability estimates will be hard to change. However, this is not necessarily incompatible with the possibility that the outcome itself (although still judged as equally likely) is now seen as being less negative by participants in the positive conditions. Further research would be needed to investigate this possibility, and the conditions necessary to modify probability.

As in any study, the present experiment has a number of limitations and the findings could be interpreted in different ways. Although intrusions were objectively categorized by an assessor, the dependence on subjective ratings to evaluate perceptions of threat raises the issue of experimenter demand, given that participants may have thought that they were expected to perceive their worry topic in a more positive way. However, on debriefing, only one participant was able to spontaneously report one of the hypotheses (although after having been told the hypotheses, a further six indicated that they had thought this is what the study was about). These seven participants were distributed across the negative verbal (3), positive verbal (2), and positive imagery (2) groups. The analyses were rerun excluding those who correctly endorsed some of the hypotheses (or all of them in the case of two participants) and the results remained unchanged. Furthermore, given the lack of similar differences in likelihood estimates, demand is unlikely to explain all the effects seen on subjective ratings.

Participants reported relatively high levels of worry, and a majority met diagnostic criteria for GAD on a questionnaire measure, but it is not clear how far the results can be generalized to clinical groups, such as those seeking treatment for GAD. In addition, the study examined only short-term effects of modifying thinking style and content of worry, so that no claims can be made as to the durability of these effects. Further studies are required before concluding either that the reduction in negative intrusions persists or that episodes of worry are also reduced, so that any therapeutic implications remain uncertain at present.

Our finding that positive mentation, whether in verbal or imagery form, led to fewer negative thoughts, reduced perceptions of threat and more positive mood, suggests that clients with GAD may benefit from prolonged practice in similar positive mentation. In a recent study where individuals with GAD practiced positive imagery or verbal thought for a week, we did indeed find evidence of reduced negative intrusions, lower anxiety, and lower trait worry, that persisted at the 1-month follow-up (Eagleson, Hayes, Mathews, Perman, & Hirsch, 2015). In clinical cognitive behavioral therapy (CBT), challenging negative automatic thoughts is usually seen as part of a process designed to elicit more positive alternatives, although recurrences of negative thought are often reported. Voluntary thought challenging requires mental effort that makes demands on limited cognitive control resources, and so may be more likely to fail under cognitive load or stress (Bowler et al., 2012). Effective methods used in the current study did not involve prolonged effortful reevaluation of negative ideas associated with worry, but rather a direct shift to imagery or positive content. It may be that this approach will prove as effective as thought challenging, and in fact similar techniques have been introduced into some CBT protocols for GAD (Borkovec & Sharpless, 2004).

One priority for future research suggested by the present results is a direct comparison of the longer term effectiveness of negative thought challenging versus more direct practice in developing relatively automated positive thought substitution, in a clinical GAD sample. The hypothesis that the effects of repeated practice in positive thought substitution may be less vulnerable to stress than thought challenging can be tested by including assessments of emotional processing bias under high and low cognitive load (for an example using the Scrambled Sentence Test, see Bowler et al., 2012).

As noted earlier, further experimental work is required to isolate more precisely the nature of the changes induced by practice in replacing worry with positive ideation. We have proposed that one critical process underlying the reduction in negative intrusions may be a reduction in the negativity of worry content. If so, the effects found here for cost, but not subjective probability, could be a consequence of different and less negative worry content being rated after positive practice than before. The subjective probability of this later (more benign) content may not change, even though the original more negative content would now be seen as less plausible. This hypothesis can be investigated by recording a detailed description of worry content both before and after training, and obtaining ratings (from participants and independent judges) of emotional negativity, cost, and probability for each description. We now predict that, after positive practice, the current content descriptions will be rated as less negative and costly, but not different in probability (thus replicating our current findings). In contrast, after positive practice the original content descriptions obtained earlier should now be rerated as being more negative and costly, but also less probable, than the current content.

In summary, the present results suggest that both thinking style and valence of worry content influence subsequent negative intrusive thoughts, which we have proposed play a critical role in the initiation of further worry episodes (Hirsch & Mathews, 2012). Although negative imagery reduced intrusions, it did not decrease the perception of threat, leading us to speculate that imagery per se does not act by changing perceived threat levels, but by blocking the priming of a wide range of threat meanings by verbal worry. Requiring participants to generate alternative positive outcomes relevant to their worries had more general effects because it decreased the perceived threat value of worry topics as well as reducing negative intrusive thoughts. These data suggest the possibility that modifying the content of worry in a more positive direction reduces the negativity of subsequent intrusive thoughts so that they are less likely to be perceived as threatening, thereby reducing the likelihood of further worry episodes.
